# Aerobic Exercise Prevents High-Fat-Diet-Induced Adipose Tissue Dysfunction in Male Mice

**DOI:** 10.3390/nu16203451

**Published:** 2024-10-11

**Authors:** Qiaofeng Guo, Nan Li, Haiyan Shi, Yanming Gan, Weiqing Wang, Jiajie Jia, Yue Zhou

**Affiliations:** 1 Department of Exercise Physiology, Beijing Sport University, Beijing 100084, China; 2 Key Laboratory of Physical Fitness and Exercise, Ministry of Education, Beijing Sport University, Beijing 100084, China

**Keywords:** adipose, inflammation, insulin resistance, macrophage, vascular smooth muscle cells

## Abstract

Background/Objectives: This study aimed to assess the effect of aerobic exercise on capillary density and vascular smooth muscle cell (VSMC) phenotype in the visceral and subcutaneous adipose tissue of high-fat-diet (HFD) mice in order to understand the mechanisms underlying improvements in insulin resistance (IR) and chronic inflammation in adipose tissue (AT). Methods: Male C57BL/6J mice were divided into HFD and normal diet groups for 12 weeks and then further split into sedentary and aerobic exercise subgroups for an additional 8 weeks. Various parameters including body weight, fat weight, blood glucose, lipid profile, insulin levels, glucose tolerance, and inflammatory cytokines were evaluated. Results: Aerobic exercise reduced HFD-induced weight gain, IR, and improved lipid profiles. HFD had a minimal effect on inflammatory cytokines except in visceral adipose tissue (VAT). IR was associated with capillary density in subcutaneous adipose tissue (SAT) and VSMC phenotype in VAT. Aerobic exercise promoted anti-inflammatory responses in VAT, correlating with VSMC phenotype in this tissue. Conclusions: Aerobic exercise can alleviate HFD-induced IR and inflammation through the modulation of VSMC phenotype in AT.

## 1. Introduction

The chronic overconsumption of energy and a sedentary lifestyle are well-established contributors to the onset of obesity-induced insulin resistance (IR), increasing the risk of metabolic disorders like type 2 diabetes and non-alcoholic fatty liver [1,2,3]. Persistent inflammation associated with obesity is frequently observed in various tissues, particularly adipose tissue (AT) [4]. Remarkably, AT, encompassing both visceral and subcutaneous fat depots, plays a crucial role in regulating metabolic processes, maintaining energy homeostasis, and influencing feeding behavior [5,6,7,8,9]. Although the distinct responses of a high-fat diet (HFD) and exercise metabolism on visceral and subcutaneous fat are known [10,11], the underlying mechanism of visceral adipose tissue (VAT) and subcutaneous adipose tissue (SAT) in IR and chronic inflammation remains unclear.

Capillaries serve as vital sites for material exchange and are crucial for the growth and function of all tissues. Proper capillarization is essential for the transport of oxygen, nutrients, and cytokines, as well as the removal of metabolites, contributing to the immune monitoring of tissues. Vascular smooth muscle cells (VSMCs), major components of arteries and veins, regulate arterial stiffness, blood pressure, and blood flow through phenotype switching. Alpha-smooth muscle actin (α-SMA) is a marker of contractile VSMCs, while osteopontin (OPN) is associated with cell growth, synthesis, proliferation, and migration. VSMCs can transition between contractile and synthetic phenotypes, influencing arterial stiffness and blood flow [12,13]. Injury to vascular endothelial cells stimulates VSMCs to release pro-inflammatory cytokines, such as interleukin-1β (IL-1β) and tumor necrosis factor-alpha (TNF-α) in insulin-resistant conditions [14,15]. However, alterations in VSMC phenotypes associated with IR in AT vessels have not been explored. Hence, we aimed to evaluate whether differences in subcutaneous and visceral fat metabolism are correlated with AT vascularity.

Regular exercise is widely recognized as an effective therapeutic approach for preventing and mitigating chronic diseases due to its positive effects on fat reduction and the enhancement of insulin sensitivity [16]. Aerobic exercise, in particular, facilitates the utilization of free fatty acids by skeletal muscle, promoting lipolysis and glucose transport. Furthermore, aerobic exercise augments the storage capacity of basal lipid droplets, reducing lipotoxicity and inflammation. Perilipin1, a lipodropin of regulating triglyceride storage and energy expenditure, indirectly explains the lipid metabolic activity and storage capacity of adipocytes [17]. Aerobic exercise improves vascular endothelial function and facilitates cytokine interactions for anti-inflammation [18]. Decreased oxygen levels in adipose tissue (AT) trigger a series of events leading to the activation of hypoxia inducible factor-1α (HIF-1α), promoting angiogenesis and adipocyte hypertrophy. Capillary density plays a crucial role in the effective communication between blood and tissues, with exercise improving SAT functional capacity for angiogenesis, which can counteract the negative effects of a high-fat diet [19]. Angiogenesis regulates the proliferation of endothelial cells, relocation to extra cellular matrix, and the formation of a lumen, as well as maintaining the circulation of the body. The proliferation of endothelial cells is regulated by the vascular endothelial growth factor (VEGF) family of growth factors [20,21]. Previous studies have shown that the amelioration of IR through aerobic exercise is associated with the inhibition of the VSMC synthesis phenotype in coronary and mesenteric arteries [22,23]. In summary, the vasculature plays a pivotal role in the anti-inflammatory process during IR development, and exercise may alleviate inflammation and IR through angiogenesis and VSMC phenotype switching in AT. Our hypothesis posits that the beneficial effects of aerobic exercise on IR and inflammation in AT are attributed to angiogenesis and the suppression of synthetic VSMCs. Therefore, our investigation explores the effects of aerobic exercise in HFD mice, unraveling the corresponding roles of inflammation and vascularity in both visceral and subcutaneous fat.

## 2. Materials and Methods

### 2.1. Animals and Diets

In this study, 5–6-week-old C57BL/6J male mice obtained from Huafukang Laboratory Animal Technology Co., Ltd. (Beijing, China), were individually free-fed (Beijing Huafukang 1025) and housed in a specific pathogen-free (SPF) facility with controlled environmental conditions (22–25 °C) and a 12 h day–night cycle in ventilated cages. After a week of acclimatization, 52 mice were single-blind randomized into two groups: HFD for 8 weeks (*n* = 30) (HFD group, 5.24 kcal/g, 60% fat, 20% protein, and 20% carbohydrate, Beijing Huafukang H10060) and a normal standard diet (*n* = 26) (NC group, 3.87 kcal/g, 2.79% fat, 23.58% protein, and 73.63% carbohydrate, Beijing Huafukang 1025). The mice were housed in groups of 3 or 4 per cage with ad libitum access to food and water. Body weight, caloric intake, and fasting blood glucose (FBG) were monitored weekly. Caloric intake is converted to calories (kcal/day/animal) based on the calorie specification of the feed mentioned above and the weight (g) consumed, as well as the number of animals kept per cage. IR was diagnosed if the area under the curve (AUC) during the glucose tolerance test (GTT) of the HFD group was statistically higher than that of the NC group. After 12 weeks of the diet, the mice underwent 8 weeks of aerobic exercise. The NC and HFD group mice were divided into two subgroups: normal sedentary (NS, *n* = 8), normal exercise (NE, *n* = 8), HFD sedentary (HS, *n* = 10), and HFD exercise (HE, *n* = 10). The animal protocol was approved by the Sports Science Experiment Ethics Committee of Beijing Sport University on 28 February 2022 (animal ethical approval reference number: 2022023A).

### 2.2. Exercise Protocol

The animals in the research followed a standard treadmill aerobic exercise protocol, with the speed gradually increasing by 3 m/min every 3 min until reaching a velocity of 15–20 m/min (corresponding to 55–65% VO2max) while maintaining the treadmill at a 0% incline [24]. Initially, the animals ran at a speed of 10–12 m/min for 20–30 min over three consecutive days to adapt to the exercise routine. Over the following 8 weeks, the mice in the exercise groups ran for 55–60 min/day, 5 days/week. In contrast, sedentary animals were placed on another stationary treadmill for 8 weeks to provide a similar environment.

### 2.3. Glucose Tolerance Test

After 12 h of fasting, the mice received an intraperitoneal injection of glucose (2 g/kg, 50% glucose) at 11 weeks and 19 weeks. Blood samples were collected from the tail vein, and blood glucose concentrations were determined at 0, 30, 60, 90, and 120 min using a glucometer (ACCU-CHEK Performa Roche, Basel, Switzerland).

### 2.4. Sample Collection

After 20 weeks of intervention, the mice were anesthetized with 2–3% isoflurane after 12 h of fasting and 48 h post-exercise training. Blood was collected from the orbit and allowed to clot at room temperature for 30 min. The clot was separated to obtain serum by centrifugation at 3500 rpm for 15 min at 4 °C. VAT and SAT were harvested from the bilateral epididymal fat pads and the abdominal subcutaneous fat of the animal, respectively. The VAT and SAT were collected and weighed. All samples were stored at −80 °C for subsequent use.

### 2.5. Biochemical Analyses

Biochemical parameters, including total cholesterol, triglyceride, high-density lipoprotein cholesterol, and low-density lipoprotein cholesterol, were measured using enzyme linked immunosorbent assay (ELSIA) kits from Njjcbio (Nanjing, China). IL-1β (Andygene Cat# AD2772Mo), TNF-α (Andygene Cat# AD3051Mo), IL-10 (Andygene Cat# AD2776Mo), and fasting insulin (Millipore Cat# EZRMI-13K, Billerica, MA, USA) were examined using enzyme-linked immunosorbent assay kits according to the manufacturer’s instructions and analyzed on Microplate Readers (BIO-RAD Cat# 168-9520, Hercules, CA, USA). The homeostasis model assessment of insulin resistance (HOMA-IR) was calculated using the equation:

HOMA-IR = [FINS (mU/L) × FBG (mmol/L)]/22.5(1)

### 2.6. Histomorphology Staining

The AT fragments measuring 0.5 × 0.5 × 0.3 cm^3^ were immersed in 4% paraformaldehyde at 4 °C for fixation. Subsequently, the tissues underwent a triple penetration with xylene and were rendered transparent during three cycles, each lasting 40 min, after dehydration. Ultimately, the tissues were immersed in paraffin and encased in embedding molds. Each fixed tissue underwent sectioning into 4 μm serial sections and dehydration. The resulting sections were placed on slides and stained with hematoxylin (Sigma-Aldrich, 03971, Saint Louis, MO, USA) for 3 min. After staining, the slides were washed with water. Then, 1% hydrochloric acid alcohol and 1% ammonia were applied for thirty seconds each, followed by another water rinse. Finally, the sections received eosin staining (Sigma-Aldrich, E4009), and the slides were secured with neutral gum overnight. Adipocyte size and number were quantified using Fiji-ImageJ (RRID:SCR_002285, Bethesda, MD, USA).

### 2.7. Immunofluorescence Analysis

Four sections from each tissue, previously fixed and deparaffinized, were submerged in EDTA antigen retrieval buffer (pH = 8.0) and exposed to microwave irradiation for 15 min. To identify the capillaries and large vessels, the sections were treated with 3% BSA (Servicebio, G5001, Wuhan, China) for 30 min, followed by an overnight incubation with primary antibodies including α-SMA (Abcam Cat# ab124964, RRID:AB_11129103, Cambridge, UK, 1:200) and CD31 (R and D Systems Cat# AF3628, RRID:AB_2161028, 1:200). After washing the membranes, the slides were exposed to anti-rabbit Cy3 (Servicebio Cat# GB21303, RRID:AB_2861435, 1:300) or anti-goat FITC (Servicebio Cat# GB22404, RRID:AB_2818950, 1:200) as secondary antibodies. Structures positive for CD31 alone classified as capillaries, while those stained with both CD31 and α-SMA were identified as large vessels [25]. Additionally, macrophage polarization (Supplemental Figure S1) was assessed by incubating with primary antibodies targeting CD11C (Proteintech Cat# 17342-1-AP, RRID:AB_2129787, Chicago, IL, USA, 1:200) and CD206 (R and D Systems Cat# AF2535, RRID:AB_2063012, 1:200) followed by secondary antibody treatment with anti-rabbit Cy3 (Servicebio Cat# GB21303, RRID:AB_2861435, 1:300) or anti-goat FITC (Servicebio Cat# GB22404, RRID:AB_2818950, 1:200). The numerical densities (scale bar = 100 μm) were calculated by observing three fields within each sample. These samples were ultimately examined using a fluorescence microscope (Leica, Wetzlar, Germany), and the mean density and areas were assessed using Fiji-ImageJ (RRID:SCR_002285).

### 2.8. Western Blotting

Total protein (20 mg) extraction was performed in 1 mM phenylmethylsulfonyl fluoride (Amresco Cat# 0332, Framingham, MA, USA) and detected using the Pierce BCA Protein Assay Kit (TDY Biotech Cat# WB0028, 1:8). After adding to SDS-PAGE sample loading buffer (TDYbio, Beijing, China, WB0031) at a final sample concentration of 4 mg/mL, the protein (20 μg) was separated on 10% SDS-PAGE gels (Sigma Cat# L4390) and transferred to nitrocellulose filter (NC) membranes (Millipore, HATF00010, Billerica, MA, USA). Proteins were blocked with 3% BSA-TBST at room temperature for 30 min and incubated with primary antibodies overnight at 4 °C, including Perilipin1 (Cell Signaling Technology Cat# 9349, RRID:AB_10829911, Danvers, MA, USA, 1:1000), HIF-1α (Immunoway Cat# YT2133, VAT 1:2000, SAT 1:4000), α-SMA (Abcam Cat# ab124964, RRID:AB_11129103, 1:100,000), and (OPN) (Abcam Cat# ab283656, RRID:AB_2894861, 1:500). The membranes were incubated with anti-rabbit (TDY Biotech Cat# S004, 1:10,000) or anti-goat (TDY Biotech Cat# S008, 1:10,000) horseradish peroxidase-linked fragment secondary antibodies for 40 min and were washed at 6 times with TBST. Protein bands were visualized by an enhanced chemiluminescence detection system (Millipore, WBKLS0500, Billerica, MA, USA), read by Total Lab Quant V11.5 (Newcastle upon Tyne, UK), and quantified using Fiji-ImageJ (RRID:SCR_002285).

### 2.9. Quantitative Real-Time Polymerase Chain Reaction Analyses

Total RNA samples containing VEGF and β-actin were extracted from AT samples using the Trizol RNA isolation reagent (Sangon Biotech, Cat# B511321, Shanghai, China) according to the manufacturer’s guidelines. For quantitative mRNA analysis, 500 ng of total RNA was reverse transcribed using reverse transcriptase (Thermo Scientific, Cat# EP0743, Waltham, MA, USA). Specific primers for VEGF and β-actin mRNA were custom-designed and produced by Sangon Biotech Co., Ltd. (Shanghai, China), through Primer Premier V5.0 (RRID: SCR_023946), with melting temperatures (Tm) set between 55 and 60 °C (the primers are listed in Supplemental Table S1). The primers were crafted ensuring a guanine–cytosine (G–C) content ranging from 20% to 80%, avoiding homopolymeric runs, and restricting the number of guanine (G) or cytosine (C) residues to two or less. Quantitative real-time polymerase chain reaction (RT-qPCR) was performed using 2X SG Fast qPCR Master Mix (Roche Cat# B639271) in LightCycler480 II (Roche, Basel, Switzerland) to detect mRNA levels. The original results were read by StepOne Plus (Applied Biosystems, Waltham, MA, USA). The relative VEGF expression was calculated using the 2^−ΔΔCT^ method [26]. 

### 2.10. Statistics

A normal distribution of data was verified with the Shapiro–Wilk test. HFD-induced IR model success rate was analyzed by One-Sample *t*-test. Repeated-measures ANOVA was used to test changes in body weight, caloric intake, and FBG. Two-tailed Student’s *t*-tests were used for comparisons between the two groups. Multi-group comparisons were performed by two-way ANOVA with Bonferroni’s multiple comparison test. Non-normal data were examined by the Scheirer Ray Hare test. Statistically significant correlations were evaluated using Pearson’s correlation coefficients. Data analysis was performed using IBM SPSS Statistics version 25.0 (RRID:SCR_019096) and RStudio (RRID:SCR_000432). Normal data of parameter tests are presented as mean ± SD, and non-normal data are presented as M (Q1, Q3). A *p*-value of <0.05 was considered statistically significant.

## 3. Results

### 3.1. Aerobic Exercise Ameliorated HFD-Induced Adiposity

**Figure 1:**
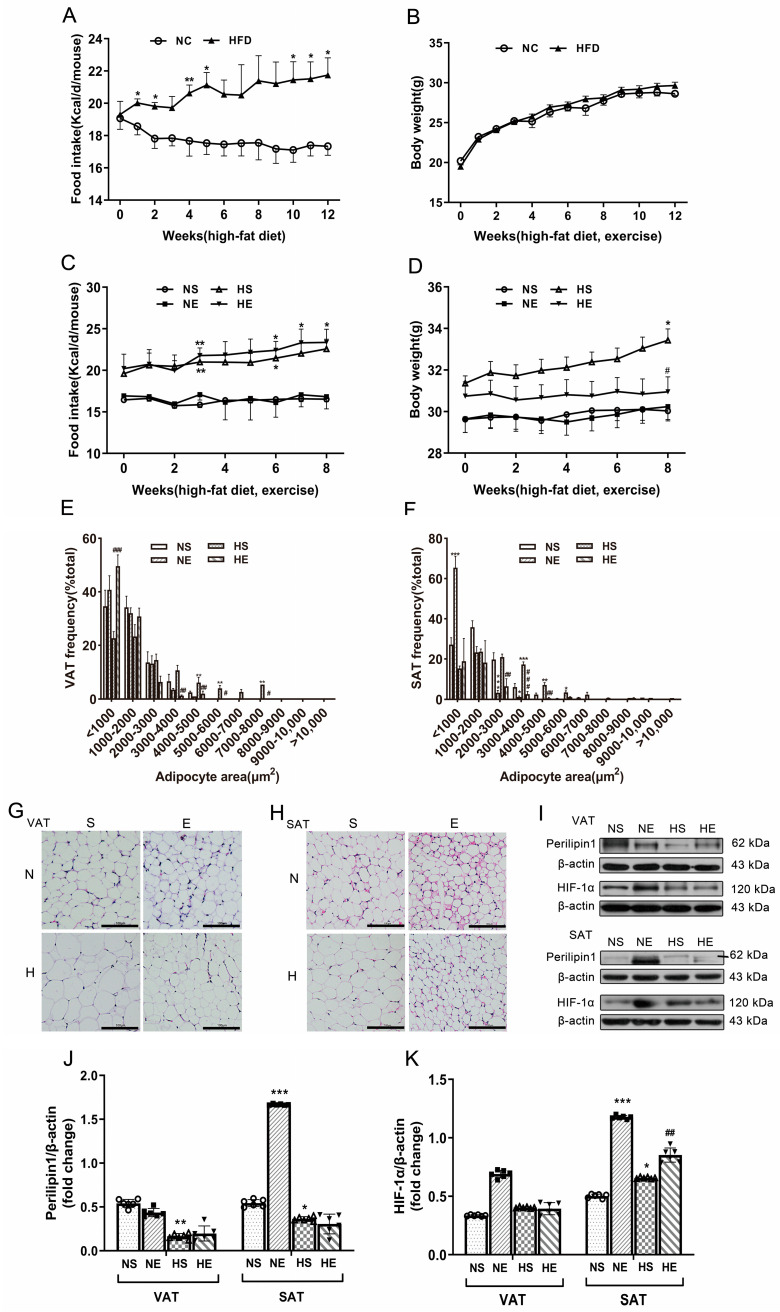
Aerobic exercise ameliorated HFD-induced adiposity. (**A**) Food intake and (**B**) body weight during 12-week feed. * *p* < 0.05, ** *p* < 0.01, *** *p* < 0.001 vs. NC. Repeated-measures ANOVA. Data are mean ± SE. *n* = 22/group. (**C**) Food intake, (**D**) body weight, and (**E**,**F**) the distribution of adipocyte sizes and histomorphology staining (scale bar = 100 μm) in (**G**) VAT and (**H**) SAT during 8 weeks of exercise. (**I**) Western blot images and quantifications of (**J**) Perilipin1 and (**K**) HIF-1α in VAT and SAT after 20 weeks. * *p* < 0.05, ** *p* < 0.01, *** *p* < 0.001 vs. NS. # *p* < 0.05, ## *p* < 0.01, and ### *p* < 0.01 vs. HS. Two-way ANOVA. Data are mean ± SE. *n* = 6–9/group. ANOVA = analysis of variance; VAT = visceral adipose tissue; SAT = subcutaneous adipose tissue.

A significant increase was noted in food intake over the 12 weeks of HFD (*p* < 0.05; Figure 1A,G). In the HS group, the distribution of expansion adipocyte (Figure 1E–H) was more pronounced, and the Perilipin1 protein expression (Figure 1I,J) was significantly decreased in both VAT and SAT compared with the NS group. Subsequent to 8 weeks of aerobic exercise, there was a decrease in body weight (*p* < 0.05; Figure 1D) accompanied by a reduction in fat weight, as well as a distribution of expansion adipocyte and serum lipid profile levels (*p* < 0.05; Figure 1E–H, Table 1 and Table 2), although no clear impact on average food intake and the expression of Perilipin1 were noted (Figure 1C,J). Besides, the HIF-1α protein expression was significantly higher because of 8-week aerobic exercise in SAT (Figure 1K). These results suggest that aerobic exercise alleviated the HFD-induced adiposity associated with VAT and SAT.

### 3.2. Aerobic Exercise Ameliorates HFD-Induced IR

**Figure 2:**
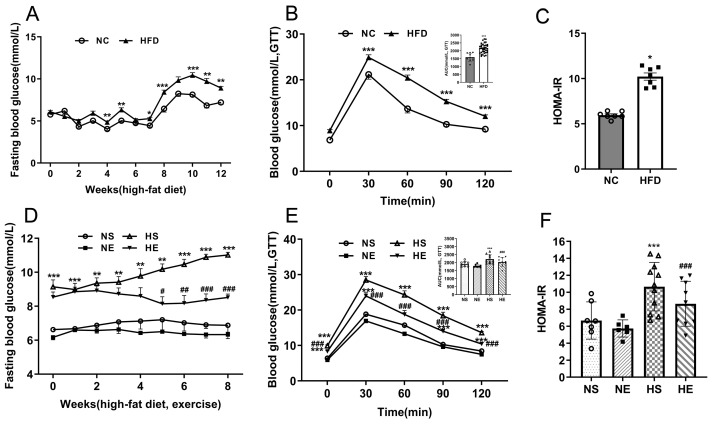
Aerobic exercise ameliorates HFD-induced IR. (**A**) FBG, (**B**) GTT, AUC of GTT, and (**C**) HOMA-IR after 12-week feed. * *p* < 0.05, ** *p* < 0.01, *** *p* < 0.001 vs. NC. Repeated-measures ANOVA. Data are mean ± SE. *n* = 22/group. (**D**) FBG, (**E**) GTT, AUC of GTT, and (**F**) HOMA-IR after 8 weeks of exercise. * *p* < 0.05, ** *p* < 0.01, *** *p* < 0.001 vs. NS. # *p* < 0.05, ## *p* < 0.01, and ### *p* < 0.01 vs. HS. Two-way ANOVA. Data are mean ± SE. *n* = 6–9/group. ANOVA = analysis of variance; FBG = fasting blood glucose; GTT = glucose tolerance test; AUC = area under the curve; HOMA-IR = homeostasis model assessment of insulin resistance.

After 12 weeks of diet intervention, IR was diagnosed if the AUC during the GTT of the HFD group was statistically higher than that of the NC group. The success rate of the IR mice model was 83.34%. The FBG levels (*p* < 0.05, *p* < 0.01), area under the curve for the glucose tolerance test (AUC_GTT_) values (*p* < 0.001, *p* < 0.001), and HOMA-IR levels (*p* < 0.05, *p* < 0.001) were impaired significantly in HFD-fed mice (Figure 2A–C). Aerobic exercise diminishes insulin resistance (*p* < 0.01) through the reduction in FBG (*p* < 0.01) and insulin concentrations, enhancing glucose tolerance (*p* < 0.01) and bolstering the capacity to react to hyperglycemia challenges at different intervals (Figure 2D–F). 

### 3.3. Effects of Aerobic Exercise on Vascular Density and VSMC Phenotype Switching of Adipose

**Figure 3:**
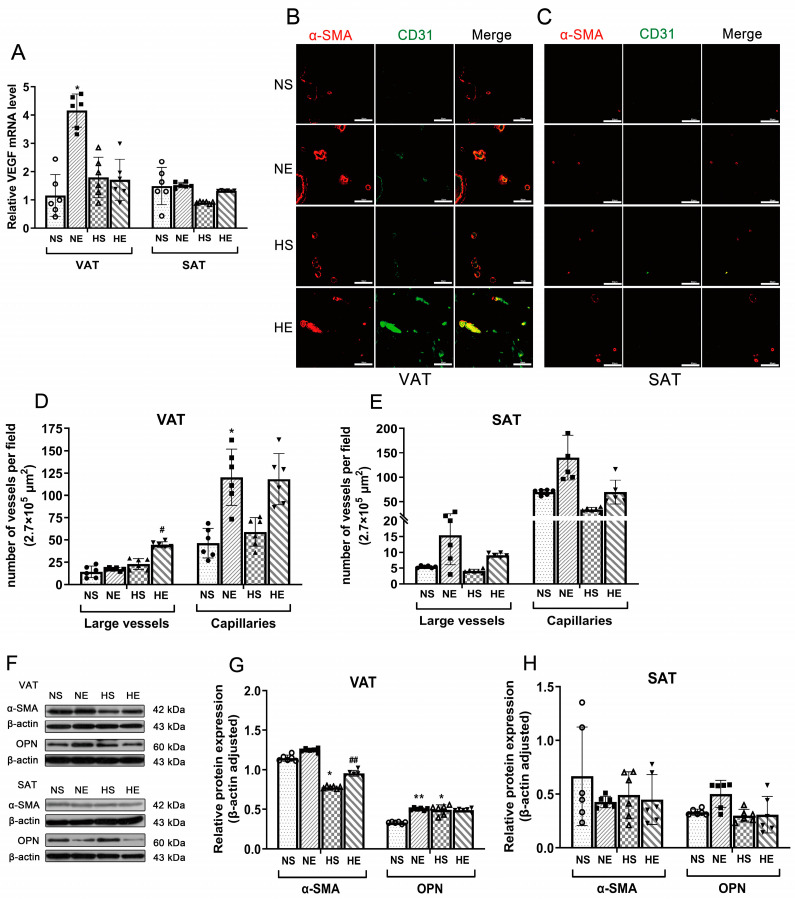
Effects of aerobic exercise on vascular density and VSMC phenotype switching of adipose. (**A**) mRNA expression of VEGF in VAT and SAT. (**B**,**C**) Immunofluorescence staining with α-SMA (red) and CD31 (green) antibodies in VAT and SAT (scale bar = 100 μm). (**D**,**E**) Density of capillary and large vessels of VAT and SAT (scale bar = 100 μm). (**F**) Western blot images and quantifications of α-SMA and OPN in (**G**) VAT and (**H**) SAT. * *p* < 0.05, ** *p* < 0.01 vs. NS. ^#^ *p* < 0.05, and ^##^ *p* < 0.01 vs. HS. Two-way ANOVA. Data are mean ± SE. *n* = 6–9/group. ANOVA = analysis of variance; VAT = visceral adipose tissue; SAT = subcutaneous adipose tissue; α-SMA = alpha-smooth muscle actin; OPN = osteopontin; VSMC = vascular smooth muscle cells.

Capillary angiogenesis and VSMC synthetic phenotype inhibition could be responsive to HFD and exercise. The large vessels, capillary density, and VEGF mRNA expression tended to increase in both the VAT and SAT of the NE group compared with the NS group (Figure 3A–E). Additionally, there was a growth of large vessels and capillary density in the HE groups in both VAT and SAT compared with the HS group (Figure 3B–E). α-SMA protein expression was suppressed by HFD (*p* = 0.001) and promoted by exercise (*p* = 0.010) in VAT (Figure 3F–H), but no significant difference was observed in α-SMA and OPN expression in SAT.

### 3.4. Correlation between IR and VSMC Phenotype Switching

We investigated the relationship between IR and angiogenesis, as well as VSMC phenotypic transformation (Table 3). The capillary density of SAT was significantly negatively correlated with FBG (*r* = −0.677, *p* = 0.016), AUC_GTT_ (*r* = −0.697, *p* = 0.012), and HOMA-IR (*r* = −0.663, *p* = 0.019) but not with VAT. The large vessel density of VAT was negatively correlated with AUC_GTT_ (*r* = −0.657, *p* = 0.020). Furthermore, α-SMA of both VAT and SAT was significantly negatively correlated with FBG (*r* = −0.967, *p* < 0.001) (*r* = −0.848, *p* < 0.001), AUC_GTT_ (*r* = −0.830, *p* < 0.001) (*r* = −0.729, *p* = 0.007), and HOMA-IR (*r* = −0.916, *p* < 0.001) (*r* = −0.896, *p* < 0.001). Additionally, HIF-1α was significantly positively correlated with capillary density (*r* = 0.683, *p* = 0.014) in VAT, large vessel density (*r* = 0.594, *p* = 0.042), and capillary density (*r* = 0.739, *p* = 0.006) in SAT.

### 3.5. Aerobic Exercise Ameliorated VAT Inflammation in HFD Mice

We evaluated chronic inflammation through the polarization of adipose macrophages and the concentration of inflammatory cytokines in the AT and serum. In our study, there were no significant differences in TNF-α and IL-1β levels after HFD or aerobic exercise (Table 4). However, during HFD, we observed a significant increase in IL-1β (*p* < 0.001; Table 5) and CD11C/CD206 ratio (*p* < 0.001; Supplemental Figure S1) and a decrease in IL-10 (*p* < 0.05). After aerobic exercise, there was a significant reduction in IL-1β levels (*p* < 0.001) and CD11C/CD206 ratio (*p* < 0.01) and an increase in IL-10 levels (*p* < 0.05) in VAT. In contrast, these changes were not observed in the SAT. Additionally, the chronic inflammation of AT is highly correlated with FBG and IR (Supplemental Table S2).

### 3.6. Correlation between Inflammation and VSMC Phenotype in HFD Mice

We further evaluated the relationship between the inflammation of serum and AT with angiogenesis and VSMC phenotypic transformation (Table 6). Moreover, α-SMA was negatively correlated with IL-1β (*r* = −0.976, *p* < 0.001) and CD11/CD206 (*r* = −0.947, *p* < 0.001) and was positively correlated with IL-10 (*r* = −0.807, *p* = 0.002) in VAT. Capillary density was significantly negatively correlated with IL-1β (*r* = −0.661, *p* = 0.007) and positively correlated with IL-10 (*r* = −0.642, *p* = 0.013) in SAT.

## 4. Discussion

It is widely acknowledged that a sedentary lifestyle coupled with a high-calorie intake establishes a detrimental cycle of obesity-related systemic IR and chronic inflammation in AT. Regular aerobic exercise has been extensively proven to contribute to fat loss, and an improvement in insulin sensitivity [20,27,28], and reduce cardiovascular risk in type 2 diabetes patients [29]. Although visceral adiposity is now recognized as a key contributor to IR, only a few studies have delved into the mechanisms by which exercise ameliorates the disparities between VAT and SAT in terms of IR and chronic inflammation. Our study not only confirms that aerobic exercise mitigates the adverse effects of an HFD on body fat, blood glucose, lipids, and IR but also highlights its association with the remodeling of the vascular network within AT.

Adipocyte growth involves two fundamental processes: hypertrophy (increase in cell size) and hyperplasia (increase in cell number). Hypertrophy is more commonly observed in the white AT of obese individuals, whereas hyperplasia is typically seen in individuals undergoing adaptive exercise training. In our study, we examined the growth and function of adipocytes in VAT and SAT following HFD and aerobic exercise. Our findings reveal that adipocyte volume expansion was more pronounced in VAT than SAT after HFD consumption. However, this expansion was significantly ameliorated by aerobic exercise. In the resting state, Perilipin inhibits triglyceride breakdown into free fatty acids in lipid droplets, promoting lipid accumulation. Conversely, Perilipin enhances lipolysis in response to triggers like cold exposure and physical activity, producing energy substrates. Our study found that HFD decreased Perilipin1 levels in VAT compared to SAT. Interestingly, Perilipin1 expression increased during aerobic exercise in SAT without dietary changes [17]. These results suggest that the detrimental effects of high-fat intake primarily impact VAT, whereas SAT acts as a protective barrier against fat transfer and visceral damage [19,30,31]. An isotopic labeling experiment could offer insights into possible variations in cell count, which is a limitation of our study. Furthermore, Perilipin1 may not provide a comprehensive evaluation of adipose function, which requires further exploration. 

In previous studies, the effect of exercise on visceral and subcutaneous fat has mainly been explored concerning fat browning and the nervous system. This study investigates the interplay between vascular density and VSMC phenotype, emphasizing its connection to local chronic inflammation and systemic IR. Immunofluorescence double staining was used to distinguish capillaries from large vessels based on their structural characteristics with endothelial cells [30,32,33]. However, it is crucial to acknowledge that this study had limitations, as it did not observe or count in a 3D capacity. Capillaries play a pivotal role in the material exchange between blood and surrounding tissues. The role of capillary neovascularization remains debated, as it can either enhance oxygen delivery to promote fat metabolism or contribute to fat storage. It is established that AT hypoxia can promote neovascularization in both positive and negative ways. On a positive note, it induces vascular neovascularization through HIF-1α, increasing the oxygen supply to ATs, promoting fat metabolism, and enhancing cellular adaptation to hypoxia [34]. Conversely, adverse effects may facilitate the transportation of lipids from the blood or other tissues to ATs for storage [20,27]. Our findings suggest that aerobic exercise promotes capillary neovascularization in SAT, correlating with improved IR. This implies that the development capillary neovascularization may confer beneficial effects through the promotion of rapid glucose and inflammatory cytokine transportation.

Regular exercise can alter VSMC phenotype and cytokine interactions towards an anti-inflammatory effect, preventing vascular remodeling, enhancing vascular function, and reducing the risk of cardiovascular disease [18]. VSMCs display phenotypic plasticity, allowing them to switch between contractile and synthetic types in response to glucolipid metabolism disorders. Healthy individuals and those engaged in adaptive sports maintain contractile VSMCs that are conducive to greater vascular elasticity and contractility, vascular substance transport, and oxygen supply. Studies indicate that moderate-intensity aerobic exercise can enhance vascular function by modifying the smooth muscle phenotype of the arteries [13,35]. Lin Zhang found that aerobic exercise intervention in insulin-resistant hypertensive mice suppresses contractile VSMCs to a synthetic phenotype [13]. Consistent with these findings, our study shows that aerobic exercise resulted in a significant increase in the number of large vessels in the VAT of obese mice, accompanied by a noteworthy upregulation of α-SMA protein expression. These findings suggest that the enhancements in large vessels in VAT with aerobic exercise mainly stem from phenotypic shifts. Furthermore, our results demonstrate a strong correlation between the large vessels improvements induced by aerobic exercise in both visceral and subcutaneous fat depots and α-SMA expression. This suggests that exercise may enhance metabolism and increase energy expenditure by stimulating HIF-1α, inducing capillary neovascularization, inhibiting VSMC proliferation and migration to ameliorate arteriosclerosis, vessel wall thickness, and blood pressure, and increasing the blood supply to AT [36,37]. This mechanism parallels the promotion of body heat production and metabolism through thermotherapy [38,39].

IR often accompanies chronic inflammation, particularly in AT. In response to a high-glucose or high-fat microenvironment, macrophages can shift toward a pro-inflammatory M1 phenotype, releasing cytokines like TNF-α and IL-1. TNF-α, acting on insulin target cells locally, directly contributes to IR [4,40]. In contrast, M2 macrophages secrete anti-inflammatory cytokines, such as IL-10. Our study observed that an HFD did not significantly alter inflammatory factors in serum and SAT. However, it notably impacts VAT, with a significant increase in IL-1β and decrease in IL-10. These changes were significantly associated with contractile VSMCs. This suggests that serum inflammatory factors reflect systemic inflammation, indicating pro or anti-inflammatory interactions in local tissues or organs. The accumulation of visceral adiposity is linked to the limited storage capacity of subcutaneous white adipocytes, leading to excess fatty acid storage around the viscera [41]. This ectopic fat accumulation can result in substance transport through the portal vein, making visceral organs more susceptible to dysfunction and inflammation. Studies indicate that for every standard deviation increase in SAT mass, the odds of developing IR decrease by approximately 48%, whereas a similar increase in VAT mass raises the odds of IR by 80% [10]. However, recent reports suggest that the activation of IR may precede chronic inflammation or even occur independently [42,43,44,45]. Our study results show that the body’s adaptation to an HFD plays a crucial role in chronic inflammation. Dietary interventions with 55% of calories from fat for 6 weeks led to a reduction in metabolic rate in the experimental group [46]. Similarly, a 6-week dietary intervention with 60% of calories from fat increased fatty acid uptake and oxidation, preventing fatty acid overload in visceral tissues [47]. These findings indicate that an HFD may activate other triggers contributing to the body’s adaptation to a high-fat intake. Further studies are needed to explore the relationship between chronic inflammation and IR by measuring inflammatory and IR-related parameters at different time intervals. Additionally, understanding how metabolic homeostasis can be achieved between tissues is essential in this context.

## 5. Conclusions

In summary, our study reaffirms the positive impact of regular aerobic exercise in mitigating the adverse effects of HFD on body fat, blood glucose, lipids, and IR, which are associated with the suppression of synthetic VSMCs in white adipose tissue and the neovascularization of capillaries in subcutaneous adipose tissue. These findings may provide a basis for a new therapeutic strategy for IR. Future studies could explore and validate specific molecular pathways to identify potential targets of VSMCs in white adipose.

## Figures and Tables

**Table 1:** Aerobic exercise attenuated HFD-induced fat weight.

Fat Weight (g)	NS	NE	HS	HE
VAT Mass	0.277 ± 0.078	0.203 ± 0.086	0.538 ± 0.138 ***	0.395 ± 0.175 ^#^
SAT Mass	0.187 ± 0.051	0.166 ± 0.179	0.356 ± 0.077 ***	0.236 ± 0.103 ^##^

*** *p* < 0.001 vs. NS. ^#^ *p* < 0.05, and ^##^ *p* < 0.01 vs. HS. Two-way ANOVA. Data are means ± SE. *n* = 6–8/group. HFD = high-fat diet; VAT = visceral adipose tissue; SAT = subcutaneous adipose tissue.

**Table 2:** Aerobic exercise attenuated HFD-induced serum lipid profile.

Serum Lipid Profile (mmol/L)	NS	NE	HS	HE
TC	2.735 ± 0.445	3.152 ± 0.558	4.157 ± 1.100 **	4.920 ± 0.216 ^#^
TG	0.681 ± 0.135	0.601 ± 0.089	0.652 ± 0.213	0.599 ± 0.066
LDL/HDL	0.044 ± 0.018	0.034 ± 0.018	0.099 ± 0.037 ***	0.622 ± 0.034 ^##^

** *p* < 0.01, *** *p* < 0.001 vs. NS. ^#^ *p* < 0.05, and ^##^ *p* < 0.01 vs. HS. Two-way ANOVA. Data are means ± SE. *n* = 6–9/group. HFD = high-fat diet; TC = total cholesterol; TG = triglyceride; LDL = low-density lipoprotein cholesterol; HDL = high-density lipoprotein cholesterol.

**Table 3:** The correlation between IR variable and VSMC phenotype.

		FBG	AUC_GTT_	HOMA-IR
VAT	Large vessel density	0.223	0.657 *	0.346
	Capillary density	−0.382	−0.125	−0.368
	α−SMA	−0.967 **	−0.830 ***	−0.916 ***
	OPN	0.276	0.324	0.330
SAT	Large vessel density	−0.452	−0.125	−0.413
	Capillary density	−0.677 *	−0.697 *	−0.663 *
	α−SMA	−0.848 **	−0.729 **	−0.896 ***
	OPN	−0.195	0.259	−0.023

*r* values’ significance set at * *p* < 0.05, ** *p* < 0.01, and *** *p* < 0.001. Pearson’s correlation analysis. Data are correlation coefficients. VAT = visceral adipose tissue; SAT = subcutaneous adipose tissue; α-SMA = alpha-smooth muscle actin; OPN = osteopontin; FBG = fasting blood glucose; GTT = glucose tolerance test; AUC = area under the curve; HOMA-IR = homeostasis model assessment of insulin resistance.

**Table 4:** Consistent levels of serum inflammatory cytokines after HFD treatment.

Inflammatory Cytokines (pg/mL)	NC	HFD
Serum TNF-α	80.125 ± 4.115	84.288 ± 8.309
Serum IL-1β	28.913 ± 0.072	29.029 ± 0.184

Two-tailed Student’s *t*-tests. Data are means ± SE. *n* = 22/group. HFD = high-fat diet; TNF-α = tumor necrosis factor-α; IL-1β = interleukin-1β.

**Table 5:** Aerobic exercise attenuated VAT inflammation in HFD mice.

Inflammatory Cytokines (pg/mL)	NS	NE	HS	HE
Serum TNF-α	73.886 ± 5.232	72.758 ± 3.514	74.606 ± 3.618	80.013 ± 5.311
Serum IL-1β	28.520 (28.394, 28.787)	28.524 (28.516, 28.667)	28.671 (28.614, 28.714)	28.660 (28.462, 28.857)
VAT IL-1β	23.505 ± 1.256	20.644 ± 0.798	49.149 ± 3.799 ***	35.799 ± 3.120 ^###^
VAT IL-10	24.587 ± 8.804	39.824 ± 7.359	12.908 ± 1.266 *	23.141 ± 3.536 ^#^
SAT IL-1β	17.973 ± 10.440	15.005 ± 7.049	19.203 ± 0.801	20.842 ± 2.220
SAT IL-10	52.773 ± 1.528	55.058 ± 1.259	49.939 ± 4.252	48.022 ± 2.053

* *p* < 0.05 and *** *p* < 0.001 vs. NS. ^#^ *p* < 0.05 and ^###^ *p* < 0.001 vs. HS. Serum IL-1β values are expressed as M (Q1, Q3) by Scheirer Ray Hare test. Other values are expressed as means ± SE by two-way ANOVA. *n* = 6–9/group. HFD = high-fat diet; TNF-α = tumor necrosis factor α; IL-1β = interleukin-1β; IL-10 = interleukin-10; VAT = visceral adipose tissue; SAT = subcutaneous adipose tissue.

**Table 6:** The correlation between inflammation and VSMC phenotype.

		Serum TNF-α	Serum IL-1β	Serum IL-10	AT IL-1β	AT IL-10	CD11C/CD206
VAT	Large vessel density	−0.171	0.012	0.156	0.392	−0.350	0.302
	Capillary density	−0.434	0.107	0.279	−0.163	0.740 **	−0.237
	α−SMA	0.273	−0.086	0.075	−0.962 ***	0.807 ***	−0.947 ***
	OPN	0.270	−0.218	−0.681 *	0.384	0.122	0.541 *
SAT	Large vessel density	−0.153	0.288	0.242	−0.456	0.526	0.385
	Capillary density	−0.237	0.090	0.119	−0.661 **	0.642 *	0.336
	α−SMA	0.162	0.340	0.379	−0.129	0.303	−0.076
	OPN	0.209	−0.326	0.035	0.687	−0.494	−0.410

*r* values’ significance set at * *p* < 0.05, ** *p* < 0.01, and *** *p* < 0.001. Pearson’s correlation analysis. Data are correlation coefficients. AT = adipose tissue; VAT = visceral adipose tissue; SAT = subcutaneous adipose tissue; α-SMA = alpha-smooth muscle actin; OPN = osteopontin.

## Data Availability

The data presented in this study are available on request from the corresponding author. The data are not publicly available due to the unavailability of the data-sharing link.

## Supplementary Materials

The following supporting information can be downloaded at https://www.mdpi.com/article/10.3390/nu16203451/s1. Figure S1: Aerobic exercise inhibited VAT macrophage M1 polarization; Table S1: Primer sequences of VEGF and β-actin; Table S2: The correlation between chronic inflammation and IR.
