# Supplementary material for: Aerobic Exercise Prevents High-Fat-Diet-Induced Adipose Tissue Dysfunction in Male Mice
Source: Nutrients. 2024 Oct 11;16(20):3451. doi: 10.3390/nu16203451 (PMC11510691; doi:10.3390/nu16203451)
Supplement: Supplementary file 1 [file nutrients-16-03451-s001.zip › Supplemental Fig. S1.pdf]

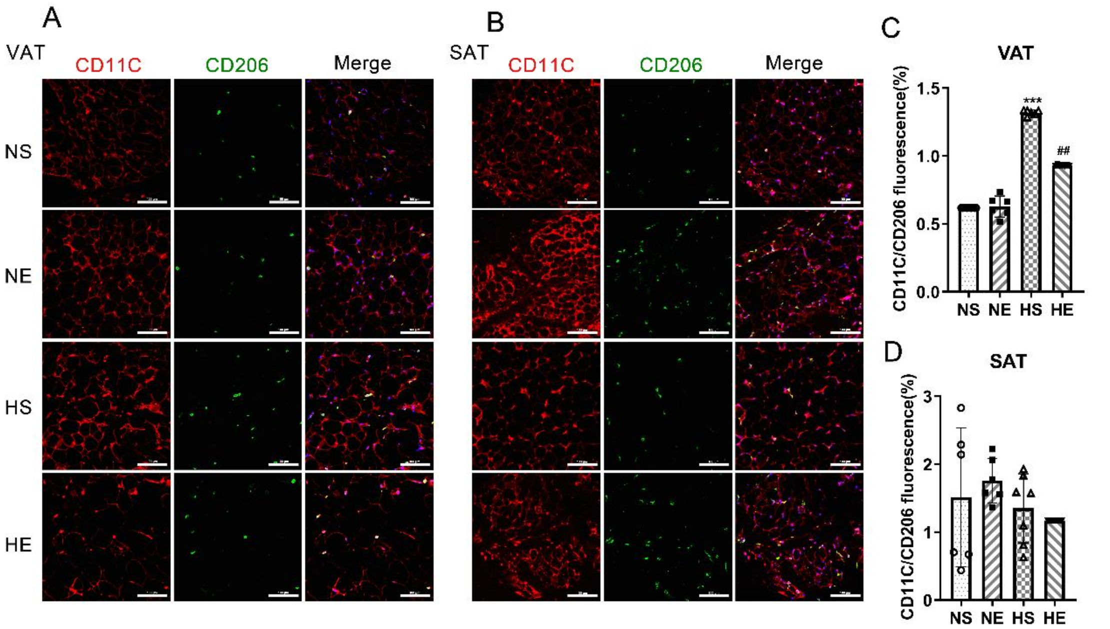

Supplemental Figure S1. Aerobic exercise inhibited VAT macrophages M1 polarization. A and B: Immunofluorescence staining with CD11C (red, macrophages M1 polarization marker) and CD206 (green, macrophages M2 polarization marker) antibodies (scale bar = 100μm) in VAT (A) and SAT (B). C and D: Mean density of CD11C/CD206 ratio in VAT (C) and SAT (D). $^{*}P < 0.05$, $^{**}P < 0.01$, $^{***}P < 0.001$ vs. NS. $^{\#}P < 0.05$, $^{\#\#}P < 0.01$ and $^{\#\#\#}P < 0.001$ vs. HS. Two-way ANOVA. Data are means ± SE. n = 6-9/group. VAT = visceral adipose tissue; SAT = subcutaneous adipose tissue.
