# Supplementary material for: Aerobic Exercise Prevents High-Fat-Diet-Induced Adipose Tissue Dysfunction in Male Mice
Source: Nutrients. 2024 Oct 11;16(20):3451. doi: 10.3390/nu16203451 (PMC11510691; doi:10.3390/nu16203451)
Supplement: Supplementary file 1 [file nutrients-16-03451-s001.zip › Supplemental Table S1.pdf]

Supplemental Table S1. Primers sequences of VEGF and β-actin

Gene	Primer Sequences (5'-3')		Product Size
VEGF	Forward Primer	CTCACCAAAGCCAGCACATAG	82bp
	Reverse Primer	TTTGTTCTGTCTTTCTTTGGTCTG	
β-actin	Forward Primer	GTGCTATGTTGCTCTAGACTTCG	174bp
	Reverse Primer	ATGCCACAGGATTCCATACC	
