# Supplementary material for: Aerobic Exercise Prevents High-Fat-Diet-Induced Adipose Tissue Dysfunction in Male Mice
Source: Nutrients. 2024 Oct 11;16(20):3451. doi: 10.3390/nu16203451 (PMC11510691; doi:10.3390/nu16203451)
Supplement: Supplementary file 1 [file nutrients-16-03451-s001.zip › Supplemental Table S2.pdf]

Supplemental Table S2. The correlation between chronic inflammation and IR

		FBG	AUC_{GTT}	HOMA-IR
VAT	IL-1β	0.962^{**}	0.491	0.498*
	IL-10	-0.678^{**}	-0.259	0.423
	CD11C/CD206	0.950**	0.471	-0.879^{*}
SAT	IL-1β	0.752^{**}	0.308	0.832^{**}
	IL-10	-0.880^{**}	-0.563^{*}	0.904^{**}
	CD11C/CD206	0.005	0.117	-0.143

r values significant set at $^{*}P < 0.05$, $^{**}P < 0.01$, $^{***}P < 0.001$. Pearson's correlation Analysis. Data are correlation coefficients. VAT = visceral adipose tissue; SAT = subcutaneous adipose tissue; α-SMA = alpha-smooth muscle actin; FBG = fasting blood glucose; GTT = glucose tolerance test; AUC = area under the curve; HOMA-IR = Homeostasis model assessment of insulin resistance.
